# Recent Developments in Microneedle Biosensors for Biomedical and Agricultural Applications

**DOI:** 10.3390/mi16080929

**Published:** 2025-08-13

**Authors:** Kazim Haider, Colin Dalton

**Affiliations:** 1 Department of Biomedical Engineering, University of Calgary, Calgary, AB T2N 1N4, Canada; cdalton@ucalgary.ca; 2 Department of Electrical and Software Engineering, University of Calgary, Calgary, AB T2N 1N4, Canada

**Keywords:** microneedles, biosensors, interstitial fluid (ISF) sensing, electrochemical sensing, smart agriculture, point-of-care diagnostics, multi-analyte detection

## Abstract

Microneedles have emerged as a versatile technology for biosensing across biomedical domains and are increasingly being explored for other applications like agriculture. This review highlights recent advancements in the development of microneedle-based biosensors in novel areas. Biomedical applications include continuous glucose monitoring, multiplexed biomarker detection beyond glucose, and numerous recent works presenting fully integrated systems comprising microneedle arrays alongside miniaturized wearable electronics. Agricultural applications largely focus on the detection of plant growth markers, hormones, and nutrient levels. Despite significant progress, challenges remain in overcoming biofouling and electrode degradation, optimizing electrode longevity for long-term (weeks to months) in situ monitoring, and creating scalable sensor fabrication processes. Additionally, there is a need for standardized mechanical and electrical testing protocols, and guidelines specifying critical performance metrics that should be reported to facilitate accurate literature comparisons. The review concludes by outlining key opportunities for future research to address these persisting challenges.

## 1. Introduction

Microneedles (MNs) have emerged as a powerful biosensing technology due to their minimally invasive nature and versatility. Typically defined as needle-like structures ranging from a few micrometers to a few millimeters in length, microneedles can penetrate biological barriers, such as the skin or the tough cell walls of plant tissues, while causing minimal tissue trauma or insertion pain. Early interest in microneedles centered on transdermal drug delivery to enhance the permeability of drugs and vaccines while mitigating many of the limitations of conventional hypodermic needles, such as insertion pain and needlephobia. Over the past two decades, the field has increasingly explored microneedles for biosensing purposes, leveraging their ability to access interstitial fluid (ISF), blood, and other biological environments in a minimally invasive manner. Recently, microneedles have been explored for use in non-biomedical applications, including agriculture and food analysis. Moreover, the field has increasingly shifted towards integrating microneedles with on-chip electronics, paving the way for compact, wearable, or point-of-care devices that can process signals on site.

Materials, fabrication methods, biocompatibility, and sensing mechanisms all play pivotal roles in defining the performance and applicability of a given microneedle biosensor. As the field of microneedle-based biosensing is growing rapidly, a comprehensive understanding of recent trends and developments is crucial. A comprehensive summary figure highlighting the different application areas of microneedle biosensors can be found in a prior review by Yang et al. (2025) [1]. This review aims to highlight the recent developments in microneedle-based biosensors, specifically electrochemical and optical biosensing in different application areas between 2024 and the first half of 2025.

## 2. Biosensing Principles and Techniques

### 2.1. Fundamentals of Biosensing

Biosensing broadly refers to the detection or continuous monitoring of biological molecules or physiological parameters using devices that integrate a biological recognition element with a signal transducer. Typically, biorecognition elements such as enzymes, antibodies, aptamers (nucleic acids), or other bioreceptors interact selectively with a target analyte such as glucose, lactate, proteins, hormones, or ions, and produce a signal that can be measured. This signal can be electrical, optical, or another quantifiable output which correlates with analyte concentration. The performance of a biosensor can be characterized by the following:

**Sensitivity**: The ability to detect low concentrations of the target analyte with minimal background noise, often defined as a ratio between the changes in the sensor response and a unit of an analyte concentration.

**Specificity**: The capacity to distinguish the target analyte from structurally similar or interfering substances in the environment.

**Reliability and stability**: Consistent performance despite variations in temperature, pH, or the composition of the biological environment.

**Response time**: The time required to achieve a stable measurable signal of the target analyte, typically the faster the better.

Sensitivity describes the magnitude of sensor output per unit analyte concentration. Highly sensitive electrochemical MN sensors often incorporate conductive materials that enhance charge transfer at the electrode interface. For example, Yang et al. (2025) reported MN sensors utilizing platinum wires electrodeposited with graphene oxide and gold nanoparticles (AuNPs) with sensitivities of 14.7 μA/μM for hydrogen peroxide detection [2]. Limit of detection (LOD) is another commonly reported metric and indicates the smallest detectable concentration above the baseline noise. Low LODs are critical for early disease diagnosis or trace analyte monitoring in biomedical or agricultural applications. Wang et al. (2024) utilized swellable hydrogel MN arrays integrated with photoelectrochemical sensors, reporting low detection limits down to femtograms per milliliter for pesticides (0.029–21 fg/mL) [3]. However, LOD is not commonly reported, as most works focus primarily on characterizing sensitivity. Response time refers to the duration required to reach a stable output upon analyte exposure, which is crucial for real-time applications. Electrochemical MN sensors typically exhibit rapid response within seconds or minutes. For instance, Tang et al. (2024) reported the near-instantaneous detection of plant hormone fluctuations within minutes of pathogen exposure using differential pulse voltammetry [4]. The stability and lifetime of MN sensors are influenced by biofouling, electrode degradation, and environmental exposure. Achieving long-term sensor stability in biological environments remains challenging. Recent strategies, such as antifouling coatings (e.g., zwitterionic polymers or epoxy propyl dimethyl ammonium chloride coatings), as demonstrated by Lv et al. (2024), improved sensor stability for extended continuous use in ISF [5].

By combining biological specificity with advancements in electronics miniaturization, modern microneedle biosensors offer rapid, accurate, and continuous monitoring capabilities, and can sample fluid directly from biological environments for on-board analysis or detect biomarkers in situ without any fluid extraction [6,7,8].

### 2.2. Common Sensing Modalities

#### 2.2.1. Electrochemical Biosensing

Electrochemical biosensors operate by measuring changes in electrical parameters like current, voltage, or impedance that occur when an analyte interacts with a functionalized electrode surface. These sensors typically utilize a three-electrode system:

**Working electrode (WE)**: Coated with or composed of the biorecognition element (e.g., enzyme, antibody, aptamer (nucleic acids)). This is where the primary electrochemical reaction occurs, producing a measurable signal proportional to the concentration of the target biomarker.

**Counter electrode (CE)**: Completes the circuit by balancing the charge passing through the working electrode.

**Reference electrode (RE)**: Maintains a stable, well-defined potential to ensure accurate and reproducible measurements at the working electrode.

Effective electrochemical biosensing relies on efficient charge transfer to the working electrode surface during the biorecognition event. For example, many recent microneedle biosensing works have focused on the detection of glucose using the glucose oxidase (GOx) enzyme [9,10,11]. This enzyme is coated on the working electrode and catalyzes the oxidation of glucose, producing gluconic acid and hydrogen peroxide, and the electrons generated are transferred to the working electrode. The generated current is proportional to the glucose concentration. This relies on having a conductive electrode surface and/or charge mediators like Prussian blue to enhance electron transfer to the WE [11,12]. Materials commonly used for the working electrode include noble metals (e.g., gold, platinum) or carbon (graphite, graphene) [2,13,14,15]. In the recent literature, there has been a shift away from fabricating the WE directly from these metals, and instead relying on processes like 3D printing, molding, or laser cutting to fabricate the base microneedle structure, followed by the application of these conductive materials through methods like sputtering or electron beam deposition [5,13,16,17]. This is largely done due to the low cost and design versatility of these methods versus traditional cleanroom-based processes that would be required for direct fabrication. Another common approach is to utilize stainless steel acupuncture or hypodermic needles, onto which conductive coatings are similarly applied, as stainless steel is a poor conductor [14,15,18,19]. The main advantage of this approach is that stainless steel needles are low-cost and widely available, though there is a possibility that they cause insertion pain and tissue trauma versus conventional microneedles due to their larger lengths. Coatings of conductive polymers such as Poly (3,4-ethylenedioxythiophene) (PEDOT) are often applied on top of the WE to enhance charge transfer [19,20]. Porous conductive polymers have also been used for enzyme entrapment, which involves coating the polymer on the WE in the presence of the enzyme so that it can be immobilized on the WE surface, thereby improving charge transfer during the biorecognition event [20].

In addition to the WE, effective electrochemical sensors require the integration of reference and counter electrodes. Reference electrodes typically utilize Ag/AgCl layers deposited via inks or pastes that have been applied through screen printing or dip coating processes onto metallic or polymeric MNs [14,21,22]. While a stable reference electrode that is unaffected by pH or temperature changes in the environment is crucial for accurate biosensing, a notable gap in the literature is the lack of studies looking at long term (>24 h) characterization studies of fabricated reference electrodes. Counter electrodes typically consist of inert conductive materials like platinum, providing stable electrical characteristics for electrochemical reactions.

Electrochemical detection methods dominate the literature due to their high sensitivity, rapid response, and suitability for miniaturization and in-field use. Amperometric electrochemical detection measures analyte-induced current changes at a fixed potential, widely used in enzyme-based sensors. Wang et al. (2024) developed an amperometric MN sensor incorporating lactate oxidase immobilized onto Prussian blue-modified stainless-steel electrodes, achieving real-time lactate monitoring in ISF with detection limits down to 15 µM [12]. Potentiometric electrochemical sensors measure voltage changes, making them suitable for ion selective measurement applications, such as monitoring potassium and sodium ions in plants. A potentiometric MN sensor developed by Wang et al. (2025) using a polymeric ion selective membrane integrated with conductive carbon ink-coated stainless steel needles demonstrated monitoring in tomato stems with detection limits in the micromolar range [15]. Electrochemical impedance spectroscopy (EIS) measures impedance changes at electrode interfaces upon analyte binding or enzymatic reactions. Dervisevic et al. (2025) used EIS with aptamer-functionalized polymeric MN arrays sputtered with gold for insulin sensing, achieving high sensitivity (65 Ω/nM) and a detection range from 0.01 to 4 nM [13].

**Figure 1:**
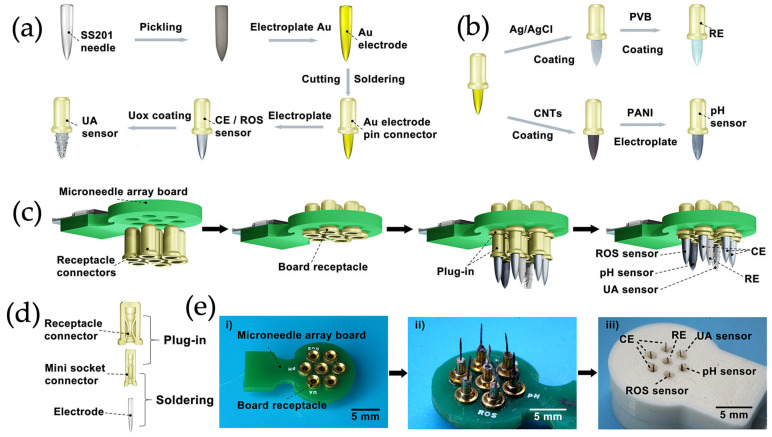
(**a**) Preparation of a stainless steel-based uric acid (UA) microneedle sensor. (**b**) Preparation of a Ag/AgCl reference electrode and pH sensor using Au-, Ag/AgCl- and carbon nanotube (CNT)-coated stainless steel 201 (SS201) microneedles. (**c**) Assembly of a microneedle-based biosensor by integrating different stainless steel-based microneedles onto a single printed circuit board. (**d**) Creation of an electrical connector on a stainless steel acupuncture needle used for biosensing. (**e**) Overview of a stainless steel acupuncture needle-based microneedle biosensor showing the (**i**) base PCB unpopulated with microneedles, (**ii**) base PCB with connected microneedles, and (**iii**) full device with CE, RE, and multiple WEs for sensing uric acid, pH, and reactive oxygen species. Reprinted (adapted) with permission from [21]. Copyright 2025 American Chemical Society.

Electrochemical microneedle biosensors offer several advantages, such as compact design, relative simplicity in signal acquisition (requiring small potentiostat circuitry), and compatibility with continuous, real-time measurements. However, challenges remain in ensuring long-term stability in complex biological environments, mitigating surface fouling, and optimizing sensor lifespan due to the depletion of the functionalizing coating [5,18]. Furthermore, since the WE, CE, and RE have different material requirements, fabricating multi-material microneedle patches can be challenging, as often each electrode is fabricated separately and assembled into a single biosensor. Figure 1 shows one example of a complex fabrication and multi-electrode assembly process utilizing stainless steel base microneedles [20].

#### 2.2.2. Optical Biosensing

Optical biosensors rely on detecting changes in light signals using methods such as absorbance, fluorescence, or colorimetry. These changes occur when a target analyte binds to a recognition element on the biosensor. When integrated with microneedles, optical sensing occurs by embedding fluorescent or colorimetric elements in or around the microneedle, or by using hollow microneedles connected to optical waveguides to sample analytes directly in tissue [23]. Common implementations involve fluorescent tags that alter their emission intensity or wavelength upon binding to the analyte, as well as colorimetric assays where a dye changes color in the presence of the target molecule [3,7,24,25]. Zhou et al. (2025) demonstrated an optical MN sensor using fluorescently labeled aptamers immobilized within methacrylated hyaluronic acid (MeHA) hydrogel MNs for cortisol monitoring [16]. The sensor detected cortisol binding via hybridization chain reaction-induced fluorescence amplification, providing a detection limit of 0.048 µM with high selectivity. Colorimetric and surface-enhanced Raman spectroscopy (SERS) methods utilize visually or spectroscopically detectable signals induced by enzymatic reactions or nanoparticle-enhanced Raman scattering. Gu et al. (2024) fabricated MN-based SERS sensors coated with gold nanoparticles, achieving highly sensitive detection of tyrosinase, a biomarker used in early melanoma screening with detection limits as low as 0.05 U/mL [26].

Li et al. (2025) developed a fluorescent microneedle biosensor for detecting biogenic amines (BAs), which are key indicators of food spoilage [27]. The sensor incorporates terbium-functionalized hydrogen-bonded organic frameworks (Tb@HOF-BPTC) into PVA hydrogel microneedles. Upon exposure to BAs, the devices exhibited fluorescence changes enabling rapid (<7 s) detection with low limits of detection (3.1–14.3 μM). The devices work with a smartphone for real-time analyte classification based on RGB fluorescence data. The hydrogel microneedle form factor enhances analyte uptake from solid or semi-solid samples and provides a portable and streamlined approach, highlighting the potential for microneedle-based fluorescence sensing platforms. Sang et al. (2023) developed a biodegradable microneedle array for continuous glucose monitoring based on fluorescence detection [28]. The microneedles are composed of silk fibroin and PVA with an embedded glucose-responsive fluorescent monomer that increases emission intensity with increasing glucose concentrations. The sensor enables minimally invasive monitoring of interstitial fluid glucose through visual or smartphone-based readouts under 405 nm excitation. In vivo experiments confirmed biocompatibility, bioresorbability, and close correlation with commercial blood glucose measurements, validating its use for real-time CGM. Other fluorescence-based microneedle biosensing works have been covered in previous literature reviews [29].

**Figure 2:**
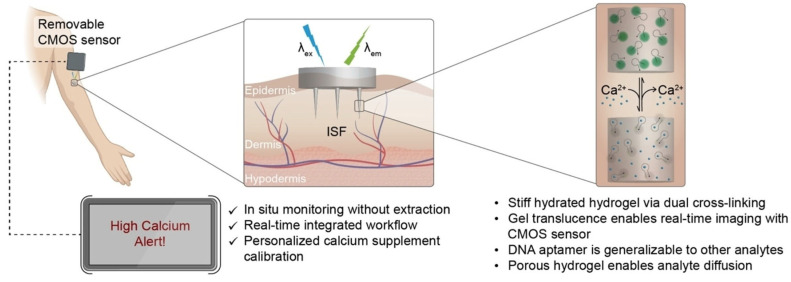
A wearable hydrogel microneedle patch with an integrated CMOS sensor for sensing Ca^2+^ through optical techniques. Adapted with permission from [24].

Optical biosensing is highly selective when utilizing fluorescently labeled antibodies or aptamers. It can provide real-time visualization and multiplexed detection if multiple wavelengths or fluorophores are used. However, drawbacks include the complexity of integrating optical components into a compact microneedle patch, susceptibility to scattering or absorption by tissue, and the need for stable and specific optical reagents that function reliably in vivo or in complex in vitro samples. Most recent articles have focused on off-board optical sensing, utilizing hydrogel microneedles to absorb analytes from interstitial fluid, followed by analysis using external lab equipment [3,7,25,30]. Recently, progress has been made by Behnam et al. (2024) towards integrating optical sensing elements directly onto the MN patch, as shown in Figure 2 [24].

## 3. Types of Microneedles for Biosensing

**Figure 3:**
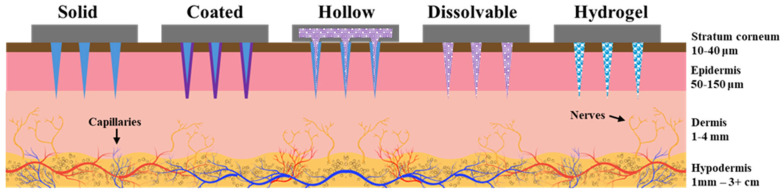
Different types of microneedles, differentiated by their structure and mode of operation.

Microneedles can be classified into four primary categories based on their structural characteristics and functionalities: solid, hollow, solid-coated, and dissolving/hydrogel [31]. Solid microneedles are commonly fabricated from metals such as stainless steel, titanium, gold, or polymers like polymethyl methacrylate (PMMA) and poly (lactic acid) (PLA). In biosensing applications, solid MNs serve primarily as electrodes that directly interface with biological fluids or tissues for measurements. As such, polymer microneedles are typically coated with conductive materials for biosensing applications. Hollow microneedles feature an internal lumen, enabling fluid extraction or analyte delivery, and are frequently employed for continuous monitoring and applications that involve on-chip or offboard analysis [6]. Conductive wires placed inside the lumens of hollow microneedles have also been used for biosensing applications [32]. Coated microneedles consist of solid or hollow MN structures modified by functional layers containing bio-recognition elements such as enzymes, antibodies, aptamers, or conductive nanomaterials, thereby enhancing biosensor selectivity and sensitivity. Dissolving and hydrogel-based microneedles are fabricated from biocompatible polymers or hydrogels that dissolve or swell upon insertion, facilitating the passive extraction or sampling of biofluids, and are mainly used in optical detection methods [24,33]. Table 1 summarizes the different types of microneedles in the context of biosensing applications, and materials and fabrication methods are further discussed in Section 4. Figure 3 shows a comparison between the different microneedle types.

## 4. Materials and Fabrication Strategies

### 4.1. Bulk Materials

The selection of bulk materials significantly impacts microneedle biosensing performance, influencing mechanical robustness, insertion performance, biocompatibility, conductivity, and fabrication complexity. Metals are frequently chosen for conductive MN biosensors due to their excellent electrical conductivity, mechanical strength, and ease of surface modification. Many recent biosensing works have also utilized polymer or stainless steel microneedles due to their relatively lower fabrication complexity. However, these require the addition of conductive surface coatings to improve their poor conductivity. Gold and gold nanoparticle (AuNP) coatings onto base microneedle structures have seen widespread use in recent works [2,9,13,21,37,38]. Polymers provide substantial flexibility in MN sensor design due to their customizable mechanical properties, biodegradability, and ease of processing through additive manufacturing. Recent works widely utilize polymeric materials like PMMA, PLA, gelatin methacrylate (GelMA), MeHA, and PDMS in MN fabrication [9,37,39,40,41].

Beyond its common use in mold fabrication, PDMS has also been used as the primary material for porous microneedles for fluid sampling and the ease of integration of PDMS-based devices with microfluidic systems. Recent studies have demonstrated PDMS-based porous microneedles for real-time biosensing in organ-on-a-chip platforms and fluid extraction interfaces in diagnostic microfluidic devices [41,42]. PDMS is one of the most widely used materials in microfluidic and biomedical device fabrication, due to its biocompatibility, gas permeability, chemical inertness, optical transparency, elasticity, and ease of molding [43]. These properties make PDMS suitable for prototyping complex microneedle geometries and integrating them with microfluidic biosensing systems. However, PDMS also has notable drawbacks, such as its intrinsic hydrophobicity, which can hinder cell adhesion and fluid flow. This can be problematic in applications requiring reliable wetting or long-term biological integration. Additionally, PDMS can absorb small hydrophobic molecules and leach uncrosslinked oligomers, potentially interfering with analyte detection or cell viability [44]. Different surface treatments can be used to mitigate this, like oxygen plasma and surfactant-based modifications to increase surface hydrophilicity.

**Figure 4:**
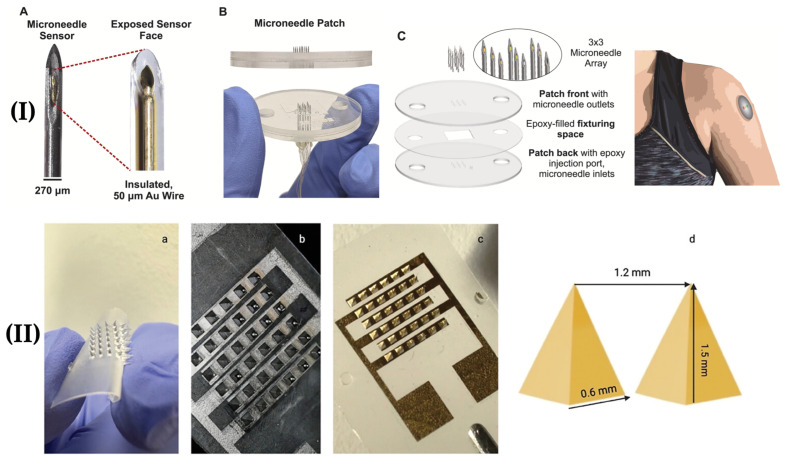
(**I**) Hollow stainless steel-based microneedles with (**A**) gold wire placed inside the lumen for enhanced conductivity, (**B**) multiple needles assembled into a patch, and (**C**) a schematic showing the patch structure and a render of it placed on a human arm. Adapted from [32] under terms of the CC-BY 4.0 license. (**II**) PLA microneedles (**a**) uncoated, (**b**) coated with chromium, (**c**) coated with gold, and (**d**) with labeled dimensions. Adapted from [40] under terms of the CC-BY 4.0 license.

Since many recent studies focus on benchtop evaluation, a gap in the current literature is around the extensive characterization of the possibility of layer delamination in microneedle designs utilizing multi-layer coatings or multi-material designs. Additionally, transverse failure load tests are often omitted, and in the works that do report this test, there is a lack of test standardization, making it difficult to compare designs across studies [45]. Figure 4 shows examples of designs utilizing stainless steel and polymer microneedles.

### 4.2. Fabrication Techniques

**Figure 5:**
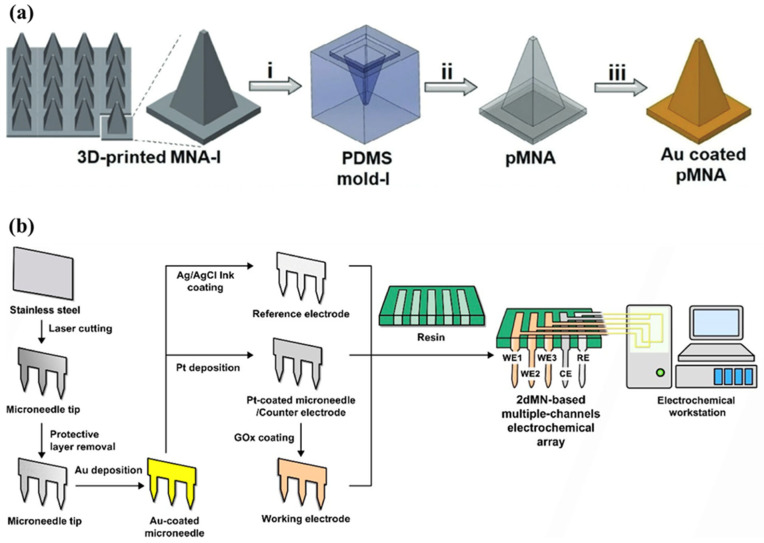
(**a**) Fabrication of a polymer microneedle array by 3D printing a master template, (i) creating a polydimethylsiloxane (PDMS) mold based on the master template, (ii) creating a polymer microneedle array (pMNA) from the PDMS mold, and (iii) coating the pMNA with gold to achieve a conductive surface for biosensing. Adapted with permission from [50]. (**b**) Fabrication of a metal microneedle array by laser cutting stainless steel base microneedles and coating them with gold, platinum or Ag/AgCl ink to form a working, counter or reference electrode. The WE, CE, and RE are fabricated separately and integrated onto a single device for biosensing. Adapted from [17] under terms of the CC-BY 4.0 license.

A variety of fabrication techniques have been utilized to achieve precise control over MN shape, size, and functionality. Photolithography combined with deep reactive ion etching (DRIE) offers high-resolution and reproducible MN structures, typically used for silicon-based MN arrays [35]. However, while traditional silicon-based MNs dominated early research, current trends lean towards utilizing these cleanroom-based processes to create highly precise, reusable master templates for polymer MN molds (e.g., PDMS molds) rather than direct silicon MN fabrication [13]. In addition to the need for simpler fabrication methods, another major reason for this shift is due to the high rigidity of silicon in contrast to the softer skin layers, which can lead to an amplified foreign body response [46]. Laser cutting and micromachining are other popular strategies that provide versatile, scalable solutions for fabricating metallic MN structures. Huang et al. (2024) created a three-electrode electrochemical sensor by laser cutting stainless steel sheets to form the microneedles, followed by depositing gold, platinum, or Ag/AgCl ink to create the working, counter and reference electrode [17]. Zhou et al. (2025) fabricated MN arrays from PMMA via computer numerical control (CNC) micromachining, creating uniform geometries robust enough for penetrating plant tissue [37]. Two-photon polymerization (2PP), a type of stereolithography printing, enables the ultra-high resolution 3D printing of polymeric MNs, allowing intricate designs and ultra-fine MN tip geometries to facilitate skin insertion. Singh et al. (2025) utilized 2PP to fabricate uniform polyurethane MN arrays with precise tip geometries, while maintaining robust mechanical properties for repeated insertions into plant tissues [38]. Kadian et al. (2025) also utilized 2PP 3D printers from Boston Micro Fabrication (BMF) to create ultrasharp microneedle arrays from photocurable polymer resins, achieving geometries suitable for the precise transdermal sensing of drugs [8]. However, due to their serial nature, direct 3D printing-based approaches may suffer from low throughput and long fabrication times. Table 2 compares recent fabrication strategies, and Figure 5 shows examples of microneedle fabrication using stainless steel and polymer base microneedles with subsequent conductive and functionalizing coatings applied.

## 5. Biomedical Applications

### 5.1. Glucose Sensing

Continuous glucose monitoring (CGM) using microneedles is a rapidly advancing area, driven by the need for minimally invasive, painless, and accurate blood glucose tracking. Recent developments include the integration of microneedle arrays with wireless sensing and telemetry hardware, and smartphone applications for real-time data visualization and remote monitoring [9]. A recent trend is the use of differential sensing, where a second sensor measures interfering substances in ISF, allowing for more accurate glucose readings [51].

Glucose detection remains a prevalent application area in the field, as it is a critical biomarker for managing diabetes, a widespread chronic disease expected to affect one in eight individuals by 2045 [56]. The need for continuous, non-invasive glucose monitoring is substantial, driving much of the research in this area. The relative simplicity of glucose detection using enzymatic methods (e.g., glucose oxidase) likely contributes to its prevalence in the literature. Furthermore, the well-established correlation between interstitial fluid glucose levels and blood glucose makes ISF a suitable target for microneedle-based glucose sensors [9,51]. Table 3 compares several recent developments in microneedle-based glucose sensors. This is not a comprehensive list, but an indication of the variety of different glucose-sensing approaches in the recent literature. Table 3 shows that impedance-based read-outs are potentially capable of rivaling amperometric sensors. Piao et al. (2024) report a 7-day mean average measurement variability of ≈4% using impedance-based sensing [52]. This approach overcomes peroxide crosstalk, a common and significant issue in amperometric glucose sensing, where applied potentials can cause co-oxidation of interfering species alongside glucose and lead to measurement errors. Additionally, there have been developments in fully closed loop diabetes management systems, with Huang et al. (2024) integrating on-patch insulin delivery alongside glucose sensing for closed-loop therapy [11].

Challenges include improving long-term stability, addressing biofouling, and further enhancing the sensitivity and selectivity of sensors, especially over long-term use (>1 week in vivo). Opportunities lie in developing integrated systems that combine sensing with drug delivery (e.g., insulin), incorporating advanced signal processing and machine learning for improved data analysis [57]. Additionally, performance indicators like in vivo lag between blood and ISF glucose levels and device longevity are still omitted by many works, hampering progress in clinical translation. Another gap in the literature involves comparisons with commercially available glucose monitors. There is also a need for standardized protocols in the field, such as standardized test methods and reporting frameworks that mandate critical parameters to be reported for every new microneedle-based sensing study, to allow for robust comparisons across the literature. The lack of standards in the microneedle field has been identified as a pressing issue in the field by the microarray patch regulatory working group (MAP-RWG), a group representing academics, industry and other stakeholders [58].

**Table 3:** Recent studies on microneedle-based glucose sensing.

MN Structure	Sensing Approach	Linear Range (mM)	Sensitivity	Validation	Ref
Stainless steel acupuncture needle/AuNP/Pt/GOx	Amperometric	0–20	0.818 µA mM^−1^	Benchtop PBS and benchtop clinical serum	[59]
Stainless steel/Au/Prussian blue/GOx/Nafion	Amperometric	0–15	77.7 nA mM^−1^ mm^−2^	Human study, benchtop PBS	[51]
3D-printed, PMMA + NSPANI/AuNPs	Amperometric	1.5–14	1.51 µA mM^−1^	In vitro ISF, gel skin model	[9]
PUA/Au/GOx	Impedance	2.8–11	−27 Ω (mg dL^−1^)	Benchtop PBS	[52]
Stainless steel/Au/CNT/Nafion/GOx/Polyurethane	Amperometric	0–30	~1500 µA mM^−1^	In vivo, closed-loop glucose measurement + insulin delivery in rats	[11]
Stainless steel/Au/Pt/GOx/Polyurethane	Amperometric	0–10	35.45–89.43 μA·mmol^−1^L^−1^	Benchtop PBS solution, rats	[22]
Photopolymer/Carbon/Prussian blue/MWCNT/Chitosan/GOx	Amperometric	0–7	2.15 µA mM^−1^	Benchtop PBS solution, agarose gel (simulated skin)	[60]

AuNP—Gold nanoparticles; GOx—glucose oxidase; PMMA—polymethyl methacrylate; NAPANI—nanostructured polyaniline; PUA—polyurethane acrylate; CNT—carbon nanotubes; MWCNT—multiwalled carbon nanotubes; PBS—phosphate-buffered saline.

### 5.2. ISF Biosensing Beyond Glucose

Beyond glucose, microneedle technology is rapidly expanding to encompass a wider array of biomarkers relevant to various health conditions. Significant progress has been made in developing sensors for uric acid, a key indicator of gout and other metabolic disorders [21,53]. These sensors often utilize electrochemical detection methods, leveraging the properties of nanomaterials like multi-walled carbon nanotubes (MWCNTs) to enhance sensitivity [21,53]. The simultaneous detection of multiple analytes is also emerging, with platforms capable of measuring glucose, uric acid, and pH from a single insertion [17,21,61]. Uric acid, urea, and pH are frequently studied because of their clinical significance and relatively straightforward detection methods. Urea levels reflect kidney function, while pH provides insights into acid–base balance [6]. Lactate is another common target, due to its role in energy metabolism and its association with various conditions, including exercise intensity and sepsis [6,61]. The relative ease of electrochemical detection for these analytes through enzyme-based approaches contributes to their prominence in the literature.

**Figure 6:**
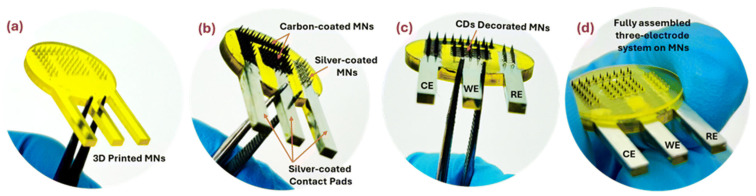
Fabrication of a chlorpromazine biosensor by (**a**) 3D printing base polymer MNs to form a WE, CE and RE, (**b**) depositing conductive carbon (WE, CE) or silver (RE) ink to improve electrical properties of each electrode, and (**c**) further modifying the WE with carbon dots (CDs) and RE with Ag/AgCl paste to form (**d**) a fully assembled microneedle biosensor. Adapted from [8] under terms of the CC-BY 4.0 license.

Beyond the commonly studied glucose, uric acid, urea, lactate, and pH, several other biomarkers are emerging as targets for microneedle-based biosensors in animal applications. Research is exploring the detection of nucleic acids, specifically cell-free DNA and RNA, for early disease diagnosis [62]. This approach leverages advancements in CRISPR-based detection technologies and offers the potential for highly sensitive and specific detection of disease-related molecules. Another area of focus is the detection of hormones such as cortisol, a key indicator of stress and adrenal function. Aptamer-based sensors have been developed for sensitive and selective cortisol detection in interstitial fluid [16]. The detection of reactive oxygen species (ROS) is also gaining traction, as ROS play a crucial role in various physiological processes and diseases like cancer [22,63]. Microneedle sensors are being developed to monitor ROS levels in real time, providing insights into oxidative stress and inflammatory responses. There is also an increasing interest in multiplexed sensing platforms capable of simultaneously detecting multiple biomarkers from a single sample [17,21,61]. This approach offers the potential for more comprehensive health assessments and personalized medicine. Additionally, Wei et al. (2025) have developed an intradermal temperature sensor leveraging microneedles, with the potential to provide in situ temperature measurements for biomedical applications or sensor measurement correction in tandem with other biosensors [46]. Figure 6 shows the fabrication of a microneedle biosensor for transdermal sensing of the schizophrenia medication chlorpromazine. Table 4 compares recent developments in microneedle-based biomarker detection beyond glucose.

### 5.3. System-Level Integration of ISF Biosensors

**Figure 7:**
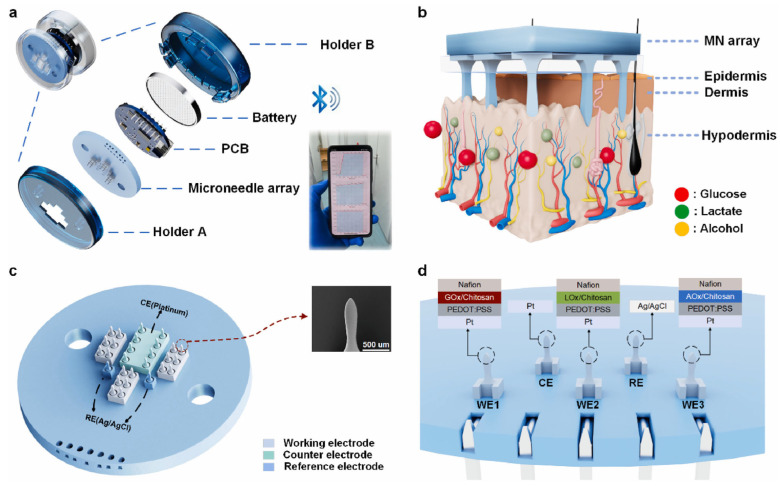
Overview of a wearable multi-analyte microneedle biosensing device showing (**a**) all device electronic components and the microneedle array, (**b**) a schematic of the microneedles interfacing with skin to detect glucose, lactate, and alcohol, (**c**) an overview of the microneedle patch with an electron microscope image of a single MN, and (**d**) the composition of the different microneedles forming the WE, CE and RE. Adapted with permission from [61].

A notable trend in the recent literature is the shift from “sensor-on-a-bench” prototypes to fully packaged devices that address wearability, power, and telemetry. Yang et al. (2024) described a wearable electrochemical microneedle device for continuous glucose monitoring, wirelessly transmitting data to a smartphone app [51]. Tawakey et al. (2024) focused on a similar device and incorporated an emergency alert system for abnormal glucose readings [9]. Zhong et al. (2024) present a wearable microneedle sensor array capable of simultaneously measuring glucose, lactate, and alcohol while also transmitting data wirelessly to a smartphone, as shown in Figure 7 [61]. Integration with smartphone apps and cloud platforms can enable remote patient monitoring and personalized medicine. Y. Liu et al. (2025) introduced a rapid, point-of-care diagnostic sensor for monkeypox virus detection, underscoring the potential of MN-based biosensors in infectious disease outbreak settings and demonstrating an extension of microneedle technology beyond metabolic markers [68]. J. Liu et al. (2024) present a microneedle patch for subcutaneous oxygen monitoring that wirelessly transmits data, eliminating the need for bulky oxygen measurement equipment and potentially enabling less invasive respiratory assessment in hospitalized patients [18]. These recent examples illustrate the versatility of microneedle-based biosensors, ranging from acute diagnostic tests to long-term physiological monitoring.

### 5.4. Trends, Challenges, and Outlooks

The majority of the ISF biosensing literature reviewed utilized amperometry, voltammetry or impedance-based sensing techniques, though optical sensing routes are emerging, driven primarily by swelling hydrogel or porous MN technologies. Looking at fabrication trends, the use of modified stainless steel acupuncture needles and hypodermic needles, and conductive material-coated polymer MNs, dominates the recent literature because these fabrication routes are low-cost, versatile, utilize off-the-shelf components, can produce mechanically robust MNs for skin insertion, and are relatively straightforward to modify for biosensing through the addition of functionalizing coatings. Multi-analyte microneedle patches are emerging but are limited by crosstalk, stable reference electrode integration, and the need for miniaturized, multichannel, low-noise interfacing electronic hardware. Some persistent technical challenges remain, as outlined in Table 5, below.

## 6. Agricultural Applications

Microneedle sensors tailored for agricultural applications are rapidly advancing to address critical needs in plant health monitoring and precision agriculture. The primary drivers that shape this research are the early detection of abiotic stress, optimization of nutrient management, pesticide residue monitoring, and real-time analysis of reactive oxygen species involved in stress signaling. For example, potassium and sodium ion dynamics are crucial indicators of plant health under salt stress conditions. Potentiometric MN sensors have been developed to non-destructively measure K^+^ and Na^+^ levels in plant sap, enabling real-time tracking from hydroponic solutions directly into plant tissues within minutes, thus significantly enhancing fertigation strategies [19]. Reactive oxygen species, especially hydrogen peroxide, play a critical role in signaling plant responses to biotic and abiotic stresses. Recent developments include hydrogel-based MN patches capable of rapid sap extraction coupled to optical colorimetric assays, allowing quick and field-deployable ROS quantification in plants such as tomatoes undergoing mechanical stress or pathogen attack [3,25,38]. Another critical area includes monitoring plant uptake of exogenous substances, such as pesticides and phenolic compounds, which has implications for food safety and quality control. Microneedle sensors employing optical detection methods have achieved ultralow detection limits for pesticides, enabling repeatable in vivo tracking and greatly simplifying field pesticide analysis protocols [3]. Similarly, MN-based colorimetric detection of total phenolic content in fruits and vegetables has shown promise for real-time nutritional assessment directly in the field [49].

**Figure 8:**
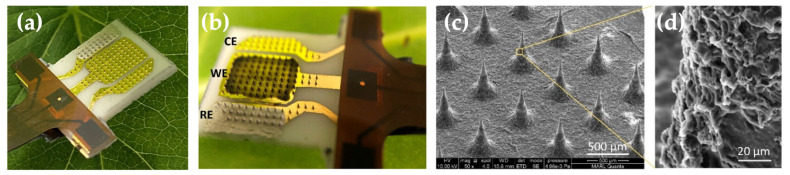
An overview of a plant biosensor (**a**) interfaced with a plant leaf, (**b**) the relative positioning of the WE, RE, and CE, (**c**) electron microscope image of individual microneedles, and (**d**) magnified view of the functionalizing coating on the WE microneedles (horseradish peroxidase/Cs-rGO). Adapted from [38] under terms of the CC-BY 4.0 license.

Singh et al. (2025) fabricated a microneedle sensor via two-photon polymerization of polyurethane, creating conical microneedles (700 μm height, 300 μm base diameter, 2 μm tip diameter), as shown in Figure 8 [38]. The working electrode comprised a 200 nm thick gold layer deposited by e-beam evaporation onto polymer microneedles, functionalized with a hydrogel composed of chitosan-reduced graphene oxide (Cs-rGO) and horseradish peroxidase for H_2_O_2_ sensing. The reference electrode utilized Ag/AgCl paste screen-printed onto MNs, and a bare gold layer on the counter-electrode MNs. This MN sensor achieved a sensitivity of 14.7 μA/μM and a detection limit of 0.06 μM for quantifying H_2_O_2_ in pathogen-infected tobacco and soybean plants. The early detection of oxidative stress can provide farmers with timely indication to implement protective measures and mitigate crop losses.

Yang et al. (2025) developed a disposable MN electrochemical sensor with platinum microneedles fabricated from 100 μm diameter platinum wires electrodeposited with graphene oxide and gold nanoparticles [2]. The CE was an unmodified Pt electrode, while the RE was a silver wire coated with Ag/AgCl. This sensor exhibited good sensitivity to H_2_O_2_ with a detection limit of 2.055 μM and excellent selectivity against common interferents found in plant fluids. The sensor monitored real-time oxidative stress responses in tomato stems following mechanical injury, highlighting its utility in precision agriculture and plant stress research.

Zhou et al. (2025) utilized CNC micromachining to fabricate PMMA microneedle arrays (conical, 800 μm height, 300 μm base diameter) [37]. The sensor comprised chromium and Au layers sputtered onto the polymer microneedles, followed by electrodeposited platinum black to enhance electrochemical sensitivity. The sensor measured electrophysiological signals associated with drought and salinity stresses, and a machine learning algorithm was used to analyze the data, achieving an accuracy rate of 99.29% in tomato seedlings. This integration of machine learning with precise microneedle technologies can enhance predictive agriculture, allowing targeted and timely interventions.

J. Zhang et al. (2024) developed an electrochemical needle sensor utilizing boron and nitrogen co-doped vertical graphene (BNVG) microelectrode arrays fabricated through electron-assisted hot-filament chemical vapor deposition [69]. The BNVG electrodes showed high sensitivity for detecting salicylic acid across concentrations of 0.5–100 µM with a detection limit of 0.14–0.18 µM. The integrated microneedle device included BNVG working electrodes, platinum counter electrodes, and tantalum reference electrodes, and was successfully applied for real-time in situ salicylic acid monitoring in cucumbers and cauliflowers. The use of a tantalum reference, a non-standard RE choice, is unique to this work and not explained. A positive correlation between salicylic acid levels and plant growth was demonstrated, showing the utility of the sensor in agriculture for precise plant health monitoring and yield prediction. Y. Zhang et al. (2025) also presented a vertical graphene-based microneedle sensor with high sensitivity and selectivity for the on-site detection of indole-3-acetic acid (IAA), an important plant growth hormone [70]. The microneedles were fabricated from vertical graphene nanosheets through chemical vapor deposition and offered large electroactive surfaces, achieving a detection limit of 0.1 µM and linear response from 0.5 to 100 µM. The sensor measured IAA levels directly in vegetable tissues and demonstrated potential for agricultural applications in monitoring plant growth regulators and improving crop management.

Chen et al. (2024) fabricated an electrochemical glucose microneedle sensor with platinum wires encapsulated within hollow polymer microneedles formed by high-resolution 3D printing [10]. The sensing interface was composed of platinum electrodes modified with gold nanoparticles, nafion, and glucose oxidase. This hollow polymer microneedle achieved a glucose detection range of 100 µM to 100 mM, with a sensitivity of 17 nA/μMcm^2^ and a detection limit of 33.3 µM. Real-time monitoring over 12 h validated the ability to detect glucose dynamics in tomato and aloe vera plants under salt stress conditions demonstrating its potential for precision crop management. Wang et al. (2024) fabricated a swellable hydrogel MN sensor from UV-crosslinked hyaluronic acid derivatives, integrated with an optical sensing mechanism using Bi_2_S_3_-Bi_2_O_3_ and modified with specific aptamers for the detection of pesticides including atrazine, carbendazim, and acetamiprid [3]. These MN arrays demonstrated low detection limits ranging from 0.029 to 21 fg/mL, enabling rapid in situ monitoring of trace pesticide levels, which is important since pesticides can influence quality, plant defense mechanisms, and crop safety for the consumer. Since previous literature reviews on the agricultural applications of microneedle biosensors, the field has rapidly expanded, with many new articles published in the 2024–2025 period, demonstrating a growing interest in the field [71,72]. Table 6 provides a summary of recent developments in the microneedle-based plant biosensing literature.

## 7. Emerging Application Areas of Conductive Microneedles

Beyond ISF sensing and agricultural applications, several novel application areas of MN biosensors have recently emerged, ranging from on-site food analysis to environmental applications. Wang et al. (2025) developed a colorimetric aptamer-based platform for detecting histamine in seafood, using a microneedle patch for rapid sample extraction [33]. The microneedles were made of polyvinyl alcohol and hyaluronic acid and optimized for swelling and histamine extraction through a two-minute press-and-peel application. The extracted histamine was detected using a DNA aptamer. The reaction produced a visible color change, providing a colorimetric readout and demonstrating the potential of microneedle-based biosensing for on-site food safety monitoring. Huang et al. (2024) developed a porous microneedle patch fabricated using plant thylakoid extract for detecting antibiotic residues in food, including fish and milk [30]. The microneedles were made from biodegradable plant-based polymers using thylakoid membranes, which produced oxygen bubbles to form porosity in the microneedles. An aptamer within the microneedles was used to selectively bind to the antibiotic netilmicin in the food samples, which were obtained from a grocery store. This antibiotic is commonly used to destroy pathogens in animal food products, but may pose a risk to human health if built up in high concentrations. The detection mechanism involved placing the microneedle patch in food samples, followed by off-board fluorescence-based quantification enabling measurements of netilmicin. Han et al. (2024) report a microneedle sensor for the in situ 3D mapping of labile copper in sediment pore water [73]. The microneedles had a nanoporous polyacrylamide matrix infused with Chelex 100 resin for high surface area and selective Cu^2+^ adsorption. The device extracted ions via passive diffusion into the porous structure, and they were then analyzed using colorimetric techniques. This work demonstrates the potential of microneedles in industrial and environmental applications.

While not a central focus of this review, microneedles are also gaining attention for integration with organ-on-a-chip and microfluidic platforms [74,75]. These hybrid systems enable dynamic, physiologically relevant environments for drug screening and disease modeling. The incorporation of microneedles into these systems allows for minimally invasive sampling, sensing, and/or targeted drug administration within engineered tissue environments, expanding their use in advanced biomedical research.

### Radiofrequency Microneedles

Radiofrequency microneedles (RF MNs) are a category of microneedles used to deliver thermal energy directly into the dermis. Unlike microneedles developed for biosensing or interstitial fluid sampling, RF MNs are primarily used for aesthetic or therapeutic applications such as acne scar treatment or skin rejuvenation through the stimulation of collagen production [76,77,78]. These devices use conductive microneedles to create microchannels in the skin while simultaneously applying radiofrequency energy to cause thermal coagulation and stimulate collagen remodeling and neoelastogenesis [76,77]. The penetration depth and thermal effects can be controlled by modulating parameters such as microneedle length, RF frequency, and the duration of the applied RF energy. Unlike laser systems that rely on chromophore absorption into skin tissue, RF-based systems are chromophore-independent, making them suitable for all skin types, including darker skin tones [76]. Despite some similarity in form to biosensing microneedles, RF MNs have not yet been explored for biochemical sensing, as their primary function is to elicit biological responses through localized thermal injury, rather than to detect or quantify biomarkers. A recent clinical trial is exploring RF microneedle therapy combined with oral isotretinoin, commonly known as the drug Accutane, for the treatment of acne [79]. While RF microneedles have demonstrated significant potential for use in dermatological therapy, they remain distinct from microneedle biosensors in both operational mechanisms and intended use cases.

## 8. Conclusions

Since 2024, the microneedles field has seen biosensing applications evolve further, from benchtop proof-of-concepts into highly integrated platforms capable of real-time monitoring in both biomedical and agricultural settings. Despite these strides, challenges remain in long-term stability, biofouling mitigation, reference electrode integration, and standardized testing and validation protocols. Future field-ready agricultural systems will require ruggedized designs, AI-driven data analytics, and mesh-network connectivity, whereas the clinical translation of biomedical microneedle sensors hinges on regulatory harmonization and long-term in vivo studies. By addressing these hurdles, microneedle biosensors are poised to transform personalized medicine, precision farming and beyond.

### 8.1. Future Outlooks for ISF Biosensing

Despite advances in microneedle-based ISF biosensors, several challenges remain. First, sensor performance often degrades over time, due to the chemical instability of sensing materials or depletion of the biorecognition element. Additionally, biofouling is a major challenge which can increase sensor impedance due to encapsulation and compromise accuracy, and high biocompatibility and accurate impedance matching in the electronics components are therefore essential to maintain reliable detection. The biocompatibility of materials is also critical for long-term wear, to prevent adverse tissue reactions such as inflammation or fibrosis [46]. Achieving consistent device performance across different manufacturing batches and user populations requires rigorous reproducibility testing. The miniaturization of the electronic components and seamless integration with microneedle electrodes pose further hurdles. Many common fabrication methods like polymer-based microneedles or stainless-steel acupuncture needles require post-processing to create a robust electronic interface. Notably, one recent study has successfully fabricated microneedles directly onto printed circuit boards via wire bonding, thereby streamlining integration with sensing electronics and paving the way for more scalable, automated manufacturing processes [20,80,81].

### 8.2. Future Outlooks for Agricultural Applications

Truly field-ready microneedle biosensors for agricultural use will need to combine mechanical robustness with sustained sensing precision under outdoor conditions that can change rapidly. Future work should focus on ruggedizing needle structures and encapsulation layers to resist rain, dust, temperature swings, and UV exposure, while implementing on-board compensation schemes (e.g., temperature and humidity calibration) to preserve accuracy over weeks or months of deployment. A 2025 IEEE technology outlook report predicts that the future of “smart agriculture” relies on the integration of AI-driven technologies [82]. Microneedles are poised to provide the data that can drive these algorithms. Scaling to large-area farms will also demand close integration with low-power electronics and wireless mesh-network architectures, enabling hundreds or thousands of nodes to stream real-time data to centralized platforms. From a biological standpoint, systematic studies are needed to optimize insertion mechanics across diverse plant tissues, like stiffer stems versus leaves, and to track any plant immune responses or biodegradation processes that might impair sensor function over time. Finally, the trajectory of these technologies will be shaped by emerging regulatory frameworks around “instrumented crops,” underscoring the importance of fully biocompatible, non-toxic materials that safeguard both plant and produce health, as well as benefitting environmental integrity.

## Figures and Tables

**Table 1:** Summary of different microneedle types in the context of biosensing applications.

MN Type	Structure and Fabrication	Biosensing Applications	Advantages	Limitations	Ref
Solid MNs	- Monolithic structure (metal, silicon, or polymer) - Fabrication via photolithography and wet/dry etching techniques (silicon), micro-milling, electrochemical etching, laser cutting, or EDM (metals), or molding techniques and 3D printing (polymers)	- Electrochemical sensing (glucose, lactate, etc.) - Biosignal detection (EMG, ECG) - Plant/environmental sensing (nutrients, salt content)	- Strong mechanical properties - Straightforward fabrication and surface functionalization - Suitable for real-time in situ measurements	- Potential for local irritation/inflammatory response - Rigid materials may not be optimal for long-term implantation due to mechanical property mismatch with soft tissues amplifying the foreign body response	[17,34]
Coated-Solid MNs	- Robust base microneedle (polymer, silicon, or metal) with a thin conductive or functional layer - Coated by methods like sputtering, electroplating, chemical vapor deposition, dip or drop coating	- Electrochemical sensors (amperometric, potentiometric, impedimetric) - Wearable diagnostics (flexible patches)	- Combines mechanical strength (core) with conductive properties (coating) - Broad biocompatibility and surface functionalization options	- Risk of delamination or corrosion of the coating causing performance degradation over time - Mechanical properties may not be as robust as a monolithic structure	[15,20]
Hollow MNs	- Central lumen for fluid extraction or injection, or housing conductive wires for sensing - Multi-step etching or molding processes using silicon, glass, metals, or polymers	- Fluid sampling and off-board or on-chip analysis - Real-time measurements in integrated lab-on-a-chip systems	- Precise, minimally invasive fluid handling - Enables continuous sampling for biomarker monitoring	- Complex fabrication to maintain both sharpness and structural integrity - Potential for lumen clogging by debris	[32,35]
Dissolvable MNs	- Water-soluble or biodegradable polymers - Fabricated via micromolding or droplet-born air blowing	- Historically limited in biosensing and primarily used for drug or vaccine delivery	- Simpler fabrication processes can facilitate mass production - Single-step application - Reduced waste and lower risk of needlestick injuries	- Unsuitable for continuous real-time or repeated sensing	[36]
Hydrogel MNs	- Swellable polymer networks (e.g., polyvinyl alcohol, polyacrylamide) - Typically fabricated via micromolding processes	- In situ analyte collection via swelling - On-chip analysis with integrated electronics - Optical detection methods	- Potential for simple visual readouts (colorimetric) - Generally soft and less traumatic to tissues	- Lower mechanical strength than solid MNs - Ensuring uniform analyte absorption and MN swelling across an array can be challenging	[24,25]

EMG—Electromyography; ECG—electrocardiography; EDM—electrical discharge machining.

**Table 2:** Comparison of recent biosensing MN fabrication strategies.

Fabrication Method	Microneedle Type	Materials	Applications	Ref
3D Printing (e.g., 2PP)	Solid, Hollow	PLA, PMMA, polymer resins	Glucose sensing, chlorpromazine sensing, pH monitoring	[8,9,47,48]
Molding	Solid	PLA, PDMS, GelMA	Vascular endothelial growth factor (VEGF) sensing, phenols detection, ascorbic acid	[13,40,49,50]
Laser Cutting or Micromachining	Solid, Hollow	Metals (stainless steel), polymers (PMMA)	Glucose sensing, pH sensing, multiplexed sensing, plant stress monitoring	[11,17,37,51]
Photolithography and wet/dry etching techniques	Solid	Silicon, noble metals (Au, Pt, etc.)	Glucose sensing, creating master molds	[52]
Inkjet Printing (onto polymer or metal base MNs)	Solid	Silver or carbon inks	Uric acid sensing, plant sensing	[53,54]
Hydrogel Casting	Hydrogel	PEGDA/pAAM, MeHA, PEDOT:PSS-based composite hydrogels	Calcium sensing, pesticide detection, hydrogen peroxide detection	[24,25,55]

PLA—Polylactic acid; PMMA—polymethyl methacrylate; PDMS—polydimethylsiloxane; PEGDA/pAAM—polyethylene glycol diacrylate/polyacrylamide; MeHA—methylated hyaluronic acid; PEDOT:PSS—poly (3,4-ethylenedioxythiophene): polystyrene sulfonate.

**Table 4:** Recent studies on MN-based biosensing in ISF beyond glucose.

Analyte(s)	MN Type or Material	Sensing Approach	Linear Range	Sensitivity	Validation	Ref
Lactate	Stainless steel needle/NiOx	Amperometric (non-enzymatic)	0.1–10 mM	0.5–3.38 μA·mM^−1^·mm^−2^	Benchtop PBS solution	[64]
Lactate	Stainless steel needles/Carbon/Chitosan/Lactate Oxidase	Amperometric	0.25–35 mM	−8.04 nA/mM	Rats	[12]
Urea, pH	Hollow MNs with MgCl_2_ lumen coating	Amperometric and potentiometric	Urea: 0–30 mM, pH: 4.7–8.6	−18.64 μA/mM (Urea), −52.82 mV/pH	Rats	[6]
Cortisol	MeHA swellable MN	Fluorescence	0.05–10 µM	4862.1 a.u./μM	Mice	[16]
Insulin	Polymer base MN + Au coating	Impedance	0.01–4 nM	65 Ω/nM	Mice	[13]
Vascular endothelial growth factor (VEGF)	PLA MN/Au Coated/functionalized with anti-VEGF antibodies	Impedance	100–1000 pg/mL	0.47 nF^−1^pg^−1^mL^−1^	Benchtop, ex vivo rat skin	[40]
Chlorpromazine	3D-printed bio resin (Boston Micro Fab)/Carbon ink	Amperometric	5–120 µM	2.65 μA/mM	Parafilm skin model + artificial ISF	[8]
Superoxide	Au MN + reduced Graphene Oxide/YHCF	Amperometric	0.304–314 μM	0.17 nA/μM	Cancer-cell spheroids	[63]
Oxygen	Au-coated acupuncture MN	Amperometric	6–150 mmHg	0.3817 μA/mmHg	Human treadmill	[18]
UA, ROS, pH	Stainless steel MNs/Au/uricase	Amperometric and potentiometric	UA: 0–0.6 mM; ROS: 0–600 µM	0.648 μA/mM (UA), 1.810 μA/mM (ROS)	Rats	[21]
UA + Dopamine (DA)	Organosilicon-modified acrylic resin/CCNT/CNT	Amperometric	UA: 5–600 µM; DA: 2–200 µM	7.13 μA μM^−1^ cm^−2^ (UA), 13.31 μA μM^−1^ cm^−2^ (DA)	Human ISF (alcohol consumption study)	[65]
UA	Polyvinyl alcohol MNs/carbon paste/uricase + antimicrobial coating	Amperometric	0.5 µM–2.5 µM and 9.6 µM–2.15 mM	0.07 µA/µM	Mice	[5]
Tyrosinase	Steel MN + Au/Ag nanoparticles	Surface-enhanced Raman spectroscopy	0.05–200 U mL^−1^	1855.90 a.u./(U/mL)	Ex vivo pig skin	[26]
K^+^, pH	Au-coated steel + nanoporous carbon	Potentiometric	K^+^: 0.1–100 mM; pH: 2–12	60 mV/decade (K^+^), −54.7 mV/decade (pH)	Benchtop PBS	[66]
Ca^2+^	PEGDA/PAA hydrogel	Fluorescence	0–2 mM	Not reported	Rats	[24]
Temperature	Acrylate resin/PEDOT:PSS	Thermistor	20–40 °C	−0.74% °C^−1^	Simulated skin, rats	[67]

NiOx—Nickel oxide; UA—uric acid; PBS—phosphate-buffered saline; ROS—reactive oxygen species; CCNT—carboxylated carbon nanotube; CNT—carbon nanotube; PEGDA/pAAM—polyethylene glycol diacrylate/polyacrylamide; PEDOT:PSS—poly (3,4-ethylenedioxythiophene): polystyrene sulfonate; YHCF—yttrium hexacyanoferrate.

**Table 5:** Persisting challenges in ISF biosensing with microneedles, recent mitigation strategies and remaining needs.

Challenge	Mitigation Strategies in the Recent Literature	Remaining Needs
Biofouling and inflammation	Antifouling coatings on MNs	Days-to-weeks scale in vivo studies
Calibration and lag time characterization	Empirical time-based corrections of measured values	Physics-based diffusion models, adaptive on-device machine learning
Reference electrode integration	Ag/AgCl-coated microneedles, solid-state quasi-references	Long-term reference electrode stability characterization after sterilization and in biological environments
Standardized testing	Recent efforts to develop standardized test methods by groups like the MAP-RWG [58]	Standardized protocols specific to the evaluation of microneedle sensors, including both electrical tests and mechanical tests like lateral failure forces.

**Table 6:** Recent literature on microneedle-based biosensing in plants.

Biomarker Detected	Microneedle Type	Microneedle Material	Sensing Approach	Ref
Indole-3-acetic acid	Solid	Graphene + Pt and Ti microelectrodes	Differential pulse voltammetry	[70]
Hydrogen peroxide	Solid	AuNPs/Graphene oxide/Pt	Chronoamperometry	[2]
Glucose	Hollow	Platinum wire + AuNPs + Nafion + GOx + PU	Amperometry	[10]
MicroRNA	Hydrogel	Methacrylated hyaluronic acid	Fluorescence	[7]
Indole-3-acetic acid and Salicylic acid	Solid	Stainless steel wire + MWCNTs	Differential pulse voltammetry and chronoamperometry	[4]
Hydrogen Peroxide	Hydrogel	PEG-crosslinked PMVE/MA	Colorimetric	[25]
K^+^ and Na^+^	Solid	Stainless steel + carbon ink (WE) + Ag/AgCl ink (RE) + ion-selective membranes	Potentiometry	[15]
pH	Solid	3D-printed polymer + Au	Potentiometry	[48]
Salicylic acid	Solid	BNVG + Pt and Ti microelectrodes	Differential pulse voltammetry	[69]
Na^+^	Solid	Stainless steel + PEDOT:PSS + ion-selective membrane	Potentiometry	[19]
Hydrogen Peroxide	Solid	Au + HRP/Cs-rGO hydrogel	Chronoamperometry	[38]

## References

[1] Yang W., Chen Y., Cheng X., Liu S., Zhu H., Hu Y. (2025). Recent Progress on the Application of Microneedles for In Situ Sampling in Surface-Enhanced Raman Scattering Detection. Biosensors.

[2] Yang X., Huo D., Tian Y., Geng X., Xu L., Zhong D., Zhou R., Xu S., Zhang Y., Sun L. (2025). AuNPs/GO/Pt Microneedle Electrochemical Sensor for in Situ Monitoring of Hydrogen Peroxide in Tomato Stems in Response to Wounding Stimulation. Anal. Bioanal. Chem..

[3] Wang J., Liu Y., Yu C., Wang X., Wang J. (2024). Swellable Microneedle-Coupled Light-Addressable Photoelectrochemical Sensor for in-Situ Tracking of Multiple Pesticides Pollution in Vivo. J. Hazard. Mater..

[4] Tang L., Li D., Liu W., Tang Y., Zhang R., Tian Y., Tan R., Yang X., Sun L. (2024). Microneedle Electrochemical Sensor Based on Disposable Stainless-Steel Wire for Real-Time Analysis of Indole-3-Acetic Acid and Salicylic Acid in Tomato Leaves Infected by *Pst* DC3000 in Situ. Anal. Chim. Acta.

[5] Lv M., Wang L., Hou Y., Qiao X., Luo X. (2025). A Wearable Antifouling Electrochemical Sensor Integrated with an Antimicrobial Microneedle Array for Uric Acid Detection in Interstitial Fluid. Anal. Chim. Acta.

[6] Li Z., Sun W., Shi Z., Cao Y., Wang Y., Lu D., Jiang M., Wang Z., Marty J.L., Zhu Z. (2025). Development of an Osmosis-Assisted Hollow Microneedle Array Integrated with Dual-Functional Electrochemical Sensor for Urea and pH Monitoring in Interstitial Fluid. Sens. Actuators B Chem..

[7] Chen L., Ding X., Dong Y., Chen H., Gao F., Cui B., Zhao X., Cui H., Gu X., Zeng Z. (2024). Integration of Catalytic Hairpin Assembly Probes into Microneedles for Detection of MicroRNA in Plants. Sens. Actuators B Chem..

[8] Kadian S., Sahoo S.S., Shukla S., Narayan R.J. (2025). Development of 3D-Printed Conducting Microneedle-Based Electrochemical Point-of-Care Device for Transdermal Sensing of Chlorpromazine. J. Mater. Chem. B.

[9] Tawakey S.H., Mansour M., Soltan A., Salim A.I. (2024). Early Detection of Hypo/Hyperglycemia Using a Microneedle Electrode Array-Based Biosensor for Glucose Ultrasensitive Monitoring in Interstitial Fluid. Lab. Chip.

[10] Chen H., Zhou S., Chen J., Zhou J., Fan K., Pan Y., Ping J. (2024). An Integrated Plant Glucose Monitoring System Based on Microneedle-Enabled Electrochemical Sensor. Biosens. Bioelectron..

[11] Huang X., Liang B., Huang S., Liu Z., Yao C., Yang J., Zheng S., Wu F., Yue W., Wang J. (2024). Integrated Electronic/Fluidic Microneedle System for Glucose Sensing and Insulin Delivery. Theranostics.

[12] Wang Q., Molinero-Fernandez Á., Wei Q., Xuan X., Konradsson-Geuken Å., Cuartero M., Crespo G.A. (2024). Intradermal Lactate Monitoring Based on a Microneedle Sensor Patch for Enhanced In Vivo Accuracy. ACS Sens..

[13] Dervisevic M., Esser L., Chen Y., Alba M., Prieto-Simon B., Voelcker N.H. (2025). High-Density Microneedle Array-Based Wearable Electrochemical Biosensor for Detection of Insulin in Interstitial Fluid. Biosens. Bioelectron..

[14] Li L., Zhou Y., Sun C., Zhou Z., Zhang J., Xu Y., Xiao X., Deng H., Zhong Y., Li G. (2024). Fully Integrated Wearable Microneedle Biosensing Platform for Wide-Range and Real-Time Continuous Glucose Monitoring. Acta Biomater..

[15] Wang Q., Molinero-Fernández Á., Acosta-Motos J.-R., Crespo G.A., Cuartero M. (2024). Unveiling Potassium and Sodium Ion Dynamics in Living Plants with an In-Planta Potentiometric Microneedle Sensor. ACS Sens..

[16] Zhou Y., He L., Zhang M., Chen M., Wu Y., Liu L., Qi L., Zhang B., Yang X., He X. (2025). An Aptamer-Responsive Microneedle Patch Sensor Platform Combining with Hybridization Chain Reaction Amplification for Detection of Steroid Hormone Cortisol in Skin Interstitial Fluid. Biosens. Bioelectron..

[17] Huang X.-S., Huang S., Zheng S.-T., Liang B.-M., Zhang T., Yue W., Liu F.-M., Shi P., Xie X., Chen H.-J. (2024). Fabrication of Multiple-Channel Electrochemical Microneedle Electrode Array via Separated Functionalization and Assembly Method. Biosensors.

[18] Liu J., Liu J., Liang Y., Yang J., Lin Y., Li Y. (2025). Microneedle-Based Electrochemical Array Patch for Ultra-Antifouling and Ultra-Anti-Interference Monitoring of Subcutaneous Oxygen. Anal. Chem..

[19] Fan C.-X., Wang Z., Wang Z.-H., Wang A.-W., Wang Z.-Y., Huang L. (2025). A Microneedle Sensor for in-Vivo Sodium Ion Detection in Plants. Anal. Chim. Acta.

[20] Haider K., Lijnse T.M., van de Panne L., Betancourt-Lee C., Lamb A., Dalton C. Wire Bonded Solid Metal Microneedles: A Versatile Platform Technology for Transdermal Drug Delivery and Biosensing. Proceedings of the Microfluidics, BioMEMS, and Medical Microsystems XXIII.

[21] Liu Z., Huang X., Liu Z., Zheng S., Yao C., Zhang T., Huang S., Zhang J., Wang J., Farah S. (2025). Plug-In Design of the Microneedle Electrode Array for Multi-Parameter Biochemical Sensing in Gouty Arthritis. ACS Sens..

[22] Huang X., Liang B., Zheng S., Wu F., He M., Huang S., Yang J., Ouyang Q., Liu F., Liu J. (2024). Microarrow Sensor Array with Enhanced Skin Adhesion for Transdermal Continuous Monitoring of Glucose and Reactive Oxygen Species. Bio-Des. Manuf..

[23] Ranamukhaarachchi S.A., Padeste C., Dübner M., Häfeli U.O., Stoeber B., Cadarso V.J. (2016). Integrated Hollow Microneedle-Optofluidic Biosensor for Therapeutic Drug Monitoring in Sub-Nanoliter Volumes. Sci. Rep..

[24] Behnam V., McManamen A.M., Ballard H.G., Aldana B., Tamimi M., Milosavić N., Stojanovic M.N., Rubin M.R., Sia S.K. (2025). mPatch: A Wearable Hydrogel Microneedle Patch for In Vivo Optical Sensing of Calcium. Angew. Chem..

[25] Wu X., Pan Y., Li X., Shao Y., Peng B., Zhang C., Zhang C., Yao S., Ping J., Ying Y. (2024). Rapid and In-Field Sensing of Hydrogen Peroxide in Plant by Hydrogel Microneedle Patch. Small.

[26] Gu Z., Zhao D., He H., Wang Z. (2024). SERS-Based Microneedle Biosensor for In Situ and Sensitive Detection of Tyrosinase. Biosensors.

[27] Li X., Zhu K., Yan B. (2025). Fluorescent Microneedle Sensors Based on Tb^3+^ @Hydrogen-Bonded Organic Frameworks for Real-Time Food Freshness Monitoring. Inorg. Chem..

[28] Sang M., Cho M., Lim S., Min I.S., Han Y., Lee C., Shin J., Yoon K., Yeo W.-H., Lee T. (2023). Fluorescent-Based Biodegradable Microneedle Sensor Array for Tether-Free Continuous Glucose Monitoring with Smartphone Application. Sci. Adv..

[29] Leanpolchareanchai J., Nuchtavorn N. (2023). Wearable Microneedle-Based Colorimetric and Fluorescence Sensing for Transdermal Diagnostics. Talanta Open.

[30] Huang R., Xu Y., Wan P., Zhu T., Heng W., Miao W. (2024). Thylakoid-Based Green Preparation of Porous Microneedles for Antibiotic Residues Detection in Food Samples. Anal. Chim. Acta.

[31] Aldawood F.K., Andar A., Desai S. (2021). A Comprehensive Review of Microneedles: Types, Materials, Processes, Characterizations and Applications. Polymers.

[32] Downs A.M., Bolotsky A., Weaver B.M., Bennett H., Wolff N., Polsky R., Miller P.R. (2023). Microneedle Electrochemical Aptamer-Based Sensing: Real-Time Small Molecule Measurements Using Sensor-Embedded, Commercially-Available Stainless Steel Microneedles. Biosens. Bioelectron..

[33] Wang W., Feng R., Wei K., Xu J., Dong W., Li J., Sun J., Wang S., Mao X. (2025). An Integrated Colorimetric Biosensing Platform Containing Microneedle Patches and Aptasensor for Histamine Monitoring in Seafood. J. Hazard. Mater..

[34] Reynoso M., Chang A.-Y., Wu Y., Murray R., Suresh S., Dugas Y., Wang J., Arroyo-Currás N. (2024). 3D-Printed, Aptamer-Based Microneedle Sensor Arrays Using Magnetic Placement on Live Rats for Pharmacokinetic Measurements in Interstitial Fluid. Biosens. Bioelectron..

[35] Zhang P., Dalton C., Jullien G.A. (2009). Design and Fabrication of MEMS-Based Microneedle Arrays for Medical Applications. Microsyst. Technol..

[36] Moawad F., Pouliot R., Brambilla D. (2025). Dissolving Microneedles in Transdermal Drug Delivery: A Critical Analysis of Limitations and Translation Challenges. J. Control. Release.

[37] Zhou J., Fan P., Zhou S., Pan Y., Ping J. (2025). Machine Learning-Assisted Implantable Plant Electrophysiology Microneedle Sensor for Plant Stress Monitoring. Biosens. Bioelectron..

[38] Singh N., Zhang Q., Xu W., Whitham S.A., Dong L. (2025). A Biohydrogel-Enabled Microneedle Sensor for In Situ Monitoring of Reactive Oxygen Species in Plants. ACS Sens..

[39] Li R., Liu Z., Xiong Y., Zhang X., Chen L., Li D., Huang C., Yu S., Jia X. (2024). A Smartphone-Enabled Colorimetric Microneedle Sensing Platform for Rapid Detection of Ascorbic Acid in Fruits. ACS Appl. Mater. Interfaces.

[40] Das R., Istif E., Cebecioglu R., Ali M., Atik Y., Dağ Ç., Celikbas E., Demirci G., Acar F., Hasanreisoğlu M. (2025). Microneedles with Interdigitated Electrodes for In Situ Impedimetric VEGF Sensing. Adv. Mater. Interfaces.

[41] Maia R., Sousa P., Pinto V., Soares D., Lima R., Minas G., Rodrigues R.O. (2024). PDMS Porous Microneedles Used as Engineered Tool in Advanced Microfluidic Devices and Their Proof-of-Concept for Biomarker Detection. Chem. Eng. J..

[42] Takeuchi K., Takama N., Kim B., Sharma K., Paul O., Ruther P. (2019). Microfluidic Chip to Interface Porous Microneedles for ISF Collection. Biomed. Microdevices.

[43] Lima R.A. (2025). The Impact of Polydimethylsiloxane (PDMS) in Engineering: Recent Advances and Applications. Fluids.

[44] Gonçalves I.M., Rodrigues R.O., Moita A.S., Hori T., Kaji H., Lima R.A., Minas G. (2022). Recent Trends of Biomaterials and Biosensors for Organ-on-Chip Platforms. Bioprinting.

[45] Haider K., Lijnse T., Shu W., O’Cearbhaill E., Dalton C. (2024). From Microchips to Microneedles: Semiconductor Shear Testers as a Universal Solution for Transverse Load Analysis of Microneedle Mechanical Performance. J. Micromechanics Microengineering.

[46] Carnicer-Lombarte A., Chen S.-T., Malliaras G.G., Barone D.G. (2021). Foreign Body Reaction to Implanted Biomaterials and Its Impact in Nerve Neuroprosthetics. Front. Bioeng. Biotechnol..

[47] Mirzajani H., Urey H. (2024). IDE-Integrated Microneedle Arrays as Fully Biodegradable Platforms for Wearable/Implantable Capacitive Biosensing. IEEE Sens. Lett..

[48] Parrilla M., Steijlen A., Kerremans R., Jacobs J., den Haan L., De Vreese J., Van Noten Géron Y., Clerx P., Watts R., De Wael K. (2024). Wearable Platform Based on 3D-Printed Solid Microneedle Potentiometric pH Sensor for Plant Monitoring. Chem. Eng. J..

[49] Li R., Liu S., Chen L., Huang C., Jia X. (2025). A Smartphone-Integrated Coated Microneedle Sensor for In-Situ Extraction and Rapid Detection of Total Phenols in Fruits and Vegetables. Anal. Sens..

[50] Dervisevic M., Harberts J., Sánchez-Salcedo R., Voelcker N.H. (2024). 3D Polymeric Lattice Microstructure-Based Microneedle Array for Transdermal Electrochemical Biosensing. Adv. Mater..

[51] Yang Y., Sheng C., Dong F., Liu S. (2024). An Integrated Wearable Differential Microneedle Array for Continuous Glucose Monitoring in Interstitial Fluids. Biosens. Bioelectron..

[52] Piao H., Choi Y.H., Kim J., Park D., Lee J., Khang D.Y., Choi H.J. (2024). Impedance-Based Polymer Microneedle Patch Sensor for Continuous Interstitial Fluid Glucose Monitoring. Biosens. Bioelectron..

[53] Nohgi T., Tu Y., Kawahira H., Kameoka J. (2025). Microneedle Uric Acid Biosensor with Graphite Ink and Electrodeposited MWCNT. IEEE Sens. J..

[54] Rosati G., Deroco P.B., Bonando M.G., Dalkiranis G.G., Cordero-Edwards K., Maroli G., Kubota L.T., Oliveira O.N., Saito L.A.M., De Carvalho Castro Silva C. (2024). Introducing All-Inkjet-Printed Microneedles for in-Vivo Biosensing. Sci. Rep..

[55] Shirzadi E., Huynh M., GhavamiNejad P., Zheng H., Saini A., Bakhshandeh F., Keyvani F., Mantaila D., Rahman F.A., Quadrilatero J. (2024). A PEDOT:PSS-Based Composite Hydrogel as a Versatile Electrode for Wearable Microneedle Sensing Platforms. Adv. Sens. Res..

[56] Global Diabetes Data Report 2000–2045. https://diabetesatlas.org/data/.

[57] Ashraf G., Ahmed K., Aziz A., Asif M., Kong J., Fang X. (2025). Microneedle Wearables in Advanced Microsystems: Unlocking next-Generation Biosensing with AI. TrAC Trends Anal. Chem..

[58] Dul M., Alali M., Ameri M., Burke M.D., Creelman B.P., Dick L., Donnelly R.F., Eakins M.N., Frivold C., Forster A.H. (2025). White Paper: Understanding, Informing and Defining the Regulatory Science of Microneedle-Based Dosage Forms That Are Applied to the Skin. J. Control. Release.

[59] Ming T., Lan T., Yu M., Duan X., Cheng S., Wang H., Deng J., Kong D., Yang S., Shen Z. (2024). A Novel Electrochemical Microneedle Sensor for Highly Sensitive Real Time Monitoring of Glucose. Microchem. J..

[60] Luo F., Li Z., Shi Y., Sun W., Wang Y., Sun J., Fan Z., Chang Y., Wang Z., Han Y. (2024). Integration of Hollow Microneedle Arrays with Jellyfish-Shaped Electrochemical Sensor for the Detection of Biomarkers in Interstitial Fluid. Sensors.

[61] Zhong G., Liu Q., Wang Q., Qiu H., Li H., Xu T. (2024). Fully Integrated Microneedle Biosensor Array for Wearable Multiplexed Fitness Biomarkers Monitoring. Biosens. Bioelectron..

[62] Yang B., Wang H., Kong J., Fang X. (2024). Long-Term Monitoring of Ultratrace Nucleic Acids Using Tetrahedral Nanostructure-Based NgAgo on Wearable Microneedles. Nat. Commun..

[63] Zouleh R.S., Rahimnejad M., Najafpour-Darzi G., Sabour D. (2025). Design of a Microneedle-Based Enzyme Biosensor Using a Simple and Cost-Effective Electrochemical Strategy to Monitor Superoxide Anion Released from Cancer Cells. Anal. Biochem..

[64] Tang Y., Huang T., Yang T., Cheng H., Cheng Y., Tsai H., Lo Y., Chen Y.S. (2025). Real-Time Lactate Detection in A Dynamic Environment Using Micrsensing Needles. Adv. Sens. Res..

[65] Zhu J., Wang W., Chen G., Gao T., Gao Z., Peng L., Wang L., Cai W. (2024). A High-Performance Wearable Microneedle Sensor Based on a Carboxylated Carbon Nanotube-Carbon Nanotube Composite Electrode for the Simultaneous Detection of Uric Acid and Dopamine. Microchem. J..

[66] Chipangura Y., Komal M., Brandao V.S.M., Sedmak C., Choi J.S., Swisher S.L., Bühlmann P., Stein A. (2024). Nanoporous Carbon Materials as Solid Contacts for Microneedle Ion-Selective Sensors. ACS Appl. Mater. Interfaces.

[67] Wei Q., Rojas D., Wang Q., Zapata-Pérez R., Xuan X., Molinero-Fernández Á., Crespo G.A., Cuartero M. (2025). Wearable 3D-Printed Microneedle Sensor for Intradermal Temperature Monitoring. ACS Sens..

[68] Liu Y., Liu J., Chen Y., Zhang G., Wang Q., Li Y. (2025). Integrated Microneedles and Hydrogel Biosensor Platform: Toward a Diagnostic Device for Collection and Dual-Mode Sensing of Monkeypox Virus A29 Protein. Anal. Chem..

[69] Zhang J., Li M., Li C., Lyu M., Xuan X., Li H. (2024). Electrochemical Needle Sensor Based on a B, N Co-Doped Graphene Microelectrode Array for the on-Site Detection of Salicylic Acid in Fruits and Vegetables. Food Chem..

[70] Zhang Y., Li M., Li H. (2025). A Vertical/Horizontal Graphene-Based Microneedle Plant Sensor for on-Site Detection of Indole-3-Acetic Acid in Vegetables. Talanta.

[71] Faraji Rad Z. (2023). Microneedle Technologies for Food and Crop Health: Recent Advances and Future Perspectives. Adv. Eng. Mater..

[72] Ece E., Eş I., Inci F. (2023). Microneedle Technology as a New Standpoint in Agriculture: Treatment and Sensing. Mater. Today.

[73] Han H., Zhang Y., Wang J., Liu R., Pan D. (2024). *In Situ* Measurement of the Three-Dimensional Distribution of Labile Copper in Sediment Pore Water Using a Microneedle Sensor with Nanoporous Structure. J. Hazard. Mater..

[74] Maia R., Carvalho V., Lima R., Minas G., Rodrigues R.O. (2023). Microneedles in Advanced Microfluidic Systems: A Systematic Review throughout Lab and Organ-on-a-Chip Applications. Pharmaceutics.

[75] Maia R.F., Machado P., Rodrigues R.O., Faustino V., Schütte H., Gassmann S., Lima R.A., Minas G. (2025). Recent Advances and Perspectives of MicroNeedles for Biomedical Applications. Biophys. Rev..

[76] Chandra S., Mysore V., Shah S., Malayanur D., Shivani S.R. (2024). Physics of Fractional Microneedle Radiofrequency—A Review. J. Cutan. Aesthetic Surg..

[77] Tan M.G., Khetarpal S., Dover J.S. (2022). Radiofrequency Microneedling. Adv. Cosmet. Surg..

[78] Zhang J., Li H., Albakr L., Zhang Y., Lu A., Chen W., Shao T., Zhu L., Yuan H., Yang G. (2023). Microneedle-Enabled Therapeutics Delivery and Biosensing in Clinical Trials. J. Control. Release.

[79] Sun Yat-Sen Memorial Hospital of Sun Yat-Sen University A Single Center, Prospective Randomized Controlled Clinical Trial on the Efficacy and Safety of Microneedle Radiofrequency Combined With Oral Isotretinoin in Moderate to Severe Acne Vulgaris, 2024. https://clinicaltrials.gov.

[80] Lijnse T., Haider K., Lee C.B., Dalton C. (2022). High Density Cleanroom-Free Microneedle Arrays for Pain-Free Drug Delivery. J. Micromech. Microeng..

[81] Haider K., Lijnse T.M., Betancourt-Lee C., Dalton C. A Method for the Rapid Fabrication of Solid Metal Microneedles. Proceedings of the Microfluidics, BioMEMS, and Medical Microsystems XXI.

[82] Abedi A., Amin M., Amirat C., Athavale J., Baker M., Byrd G., Chard K., Coughlin T., Hajj I.E., Faraboschi P. Technology Predictions 2025. https://ieeecs-media.computer.org/media/tech-news/tech-predictions-report-2025.pdf.

